# The association between triglyceride-glucose index and related parameters and risk of cardiovascular disease in American adults under different glucose metabolic states

**DOI:** 10.1186/s13098-024-01340-w

**Published:** 2024-05-17

**Authors:** Yuansong Zhuang, Liliang Qiu, Dongjian Han, Zhentao Qiao, Fuhang Wang, Qingjiao Jiang, Quanxu An, Yuhang Li, Jiahong Shangguan, Xuanye Bi, Deliang Shen

**Affiliations:** 1 Cardiology Department, First Affiliated Hospital of Zhengzhou University, Henan, China; 2 Department of Respiratory Medicine, First Affiliated Hospital of Zhengzhou University, Henan, China; 3 Department of Vascular and Endovascular Surgery, First Affiliated Hospital of Zhengzhou University, Henan, China

**Keywords:** Triglyceride glucose index (TyG), Cardiovascular disease, National health and nutrition examination survey (NHANES), Different glucose metabolic states

## Abstract

**Background:**

Cardiovascular disease (CVD) encompasses an array of cardiac and vascular disorders, posing a significant threat to global health. It remains unclear whether there exists an association between triglyceride-glucose index (TyG) and its derived indices and the incidence of cardiovascular disease, and in particular, the strength of the association in populations with different glucose metabolisms is not known.

**Methods:**

Data extracted from the National Health and Nutrition Examination Survey (NHANES) covering the period from 1999 to 2020, involving a cohort of 14,545 participants, were leveraged for the analysis. Statistical assessments were executed utilizing R software, employing multivariable logistic regression models to scrutinize the correlation between TyG and its associated parameters with the incidence of cardiovascular disease across diverse glucose metabolism categories. Interaction analyses and restricted cubic splines were applied to evaluate potential heterogeneity in associations and investigate the link between TyG and its derivatives with the occurrence of cardiovascular disease. Furthermore, receiver operating characteristic curves were constructed to evaluate the extent of variability in the predictive performance of TyG and its derived parameters for cardiovascular disease across distinct glucose metabolic statuses.

**Results:**

This study found that TyG and its related parameters were differentially associated with the occurrence of cardiovascular disease in different glucose metabolic states. Curvilinear correlations were found between TyG in the IFG population and TyG-WC, TyG-BMI, and TyG-WHtR in the impaired glucose tolerance (IGT) population with the occurrence of cardiovascular disease. In addition, the introduction of TyG and its derived parameters into the classical Framingham cardiovascular risk model improved the predictive performance in different glucose metabolism populations. Among them, the introduction of TyG-WHtR in the normal glucose tolerance (NGT), impaired fasting glucose (IFG), IFG & IGT and diabetes groups and TyG in the IGT group maximized the predictive power.

**Conclusions:**

The findings provide new insights into the relationship between the TyG index and its derived parameters in different glucose metabolic states and the risk of cardiovascular disease, offering important reference value for future clinical practice and research. The study highlights the potential for improved risk stratification and prevention strategies based on TyG and its derived parameters.

**Supplementary Information:**

The online version contains supplementary material available at 10.1186/s13098-024-01340-w.

## Introduction

Cardiovascular disease (CVD) encompasses an array of cardiac and vascular disorders, posing a significant threat to global health [1]. Established risk factors such as male gender, obesity, dyslipidemia, hypertension, diabetes, and smoking are closely associated with the occurrence and progression of cardiovascular diseases [2]. Despite substantial advances in prevention and treatment strategies, CVD remains a predominant cause of global mortality and disability [3]. Given the escalating public health burden imposed by CVD, understanding clinical predictive factors for the preclinical stages of cardiovascular disease is paramount for preventing disability, dependency, and preserving quality of life.

Diabetes mellitus is a cardiovascular disease equivalent, sharing numerous common risk factors [4]. Substantial research evidence indicates a close association between diabetes and adverse outcomes in cardiovascular disease patients, particularly in insulin-resistant type diabetes [5]. In recent years, attention from researchers has shifted from diabetes to the earlier stage of insulin resistance, aiming to implement necessary measures to reduce the disability and mortality rates of cardiovascular disease at an earlier stage. However, current clinical assessments of insulin resistance, such as the hyperinsulinemic-euglycemic clamp and intravenous glucose tolerance test, are constrained by their high cost and complex procedures [6, 7].

The triglyceride-glucose index (TyG) serves as a readily measurable biochemical marker, initially introduced in 2008 as a tool for identifying insulin resistance in ostensibly healthy individuals [8]. Subsequent studies have further demonstrated that TyG exhibits superior or comparable performance to other indicators, such as HOMA-IR, in evaluating insulin resistance [9]. Body mass index (BMI), waist circumference (WC), and waist-to-height ratio (WHtR) are obesity indices associated with increased insulin resistance and cardiovascular disease risk [10–12]. Derived composite parameters, including TyG-WC, TyG-BMI, and TyG-WHtR, seem to more practically reflect a patient’s IR status and offer greater cost-effectiveness, exhibiting high diagnostic value in previous studies [10, 13]. However, it remains unclear whether there exists an association between TyG and its derived indices and the incidence of cardiovascular disease, and in particular, the strength of the association in populations with different glucose metabolisms is not known.

Therefore, this study aims to investigate the relationship between TyG and its derived indices and the occurrence of cardiovascular disease in different glucose metabolic states using data from the National Health and Nutrition Examination Survey (NHANES). By undertaking this research, we endeavor to provide additional evidence concerning the practical application of TyG and its derived indices in the real world, aiming to offer more reliable indicators for assessing individual cardiovascular disease risk and promoting early intervention and preventive measures for cardiovascular disease.

## Materials and methods

### Study design and population

The National Health and Nutrition Examination Survey (NHANES) is a meticulously designed and comprehensive survey jointly developed by the Centers for Disease Control and Prevention (CDC) and the National Center for Health Statistics (NCHS). It aims to gather nationally representative data from non-institutionalized civilian populations in the United States through a complex, stratified, multistage probability cluster sample survey design. This ongoing survey conducts independent cross-sectional assessments every two years. NHANES employs standardized household interviews, health examinations at mobile examination centers, and laboratory tests to evaluate participants’ medical and physical conditions.

**Fig 1:**
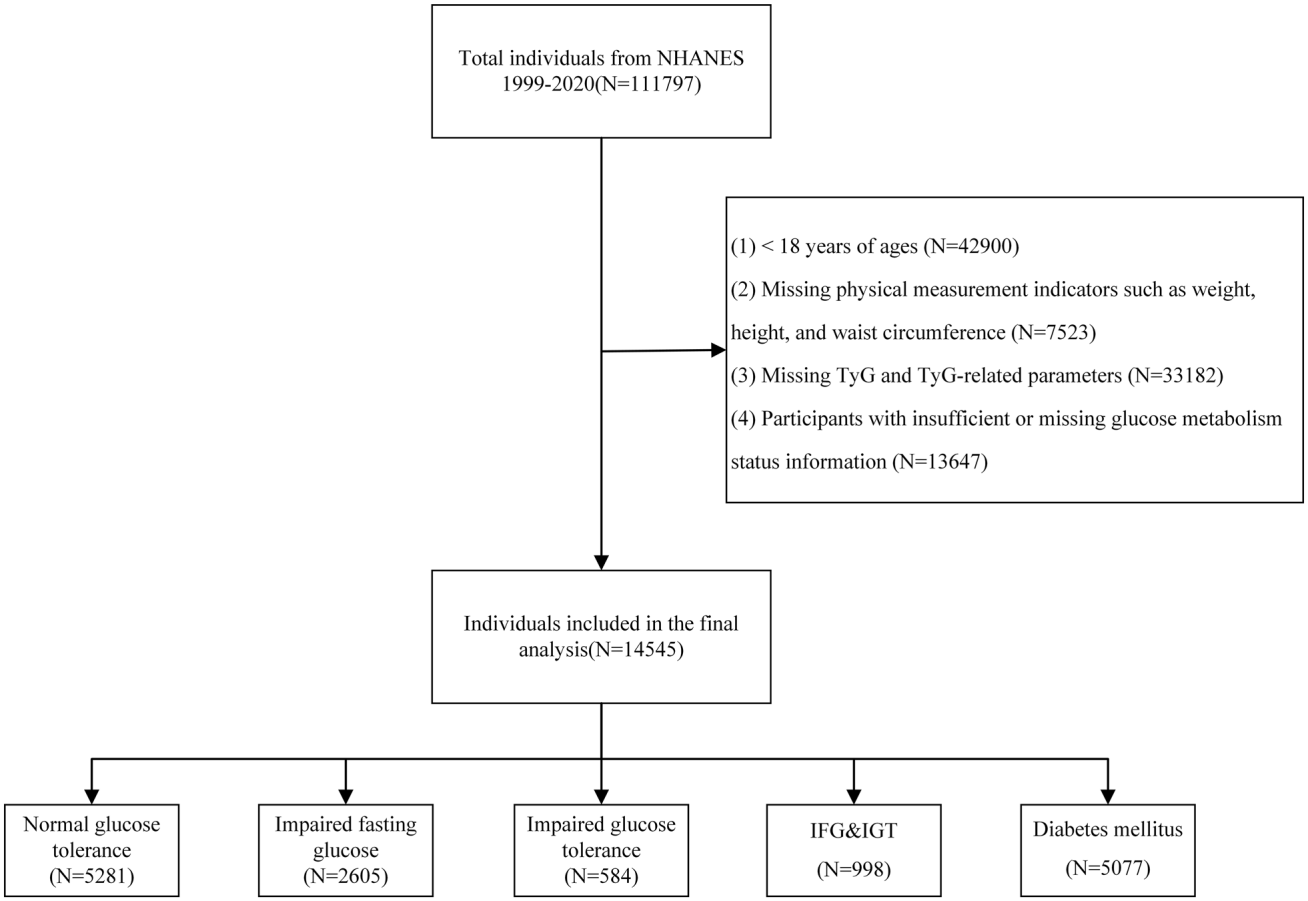
Flow chart for study population selection

In this study, we selected data from 11 NHANES survey cycles spanning from 1999 to 2020, resulting in a total of 111,797 anonymous records. The following exclusion criteria were applied: (1) individuals under 18 years of age, (2) those with missing body measurements such as weight, height, or waist circumference, (3) participants with incomplete TyG data or missing derived TyG data, and (4) individuals with insufficient or missing information on glucose metabolism. Ultimately, 14,545 screened participants were included in this study. For detailed information on the selection process, please refer to Fig. 1. The NHANES research obtained ethical approval from the Institutional Review Board at the National Center for Health Statistics and secured written informed consent from all research participants, ensuring their voluntary participation in the survey.

### Laboratory tests and clinical data

After fasting for 12 h, NHANES participants provided blood samples at standardized mobile examination centers. Whole blood cell counts and biochemical indicators were determined using automated hematological analysis equipment. The collected blood samples underwent comprehensive laboratory testing recommended by NHANES, including white blood cells, hemoglobin, platelets, fasting plasma glucose (FPG), alanine aminotransferase (ALT), aspartate aminotransferase (AST), alkaline phosphatase (ALP), total bilirubin, creatinine, uric acid, blood urea nitrogen, triglycerides, low-density lipoprotein (LDL), high-density lipoprotein (HDL), C-reactive protein (CRP), and glycated hemoglobin levels. For detailed information on the specific testing methods used, please visit the NHANES website (https://wwwn.cdc.gov/nchs/nhanes/AnalyticGuidelines.aspx).

Clinical information about the participants, including age, gender, education level, race, poverty income ratio (the ratio of family income to the local poverty line), smoking consumption, and alcohol consumption, was obtained from the original database. In addition, measurements such as height (cm), weight (kg), waist circumference (cm), waist-to-height ratio, and body mass index (BMI) were recorded. Disease status, including diabetes, hypertension, chronic obstructive pulmonary disease (COPD), cardiovascular disease, and related medication use, was also documented. Education level was categorized as less than high school, high school, or above. Racial/ethnic categories included non-Hispanic White, non-Hispanic Black, Mexican American, and other. Poverty income ratio was classified as poor (PIR < 1.3), middle income (PIR 1.3–3.5), and affluent (PIR > 3.5). Smoking status was categorized as current smoker (having smoked at least 100 cigarettes in lifetime and currently smoking), former smoker (having smoked at least 100 cigarettes in lifetime but not currently smoking), and never smoker (not meeting the aforementioned criteria). Alcohol consumption was categorized as never, mild, moderate, and heavy drinkers, following the same classification as previous studies [14]. The Healthy Eating Index (HEI) served as an indicator for assessing dietary quality, obtained by calculating individual scores for 12 dietary components (e.g., total vegetables, whole grains, milk) to yield a total score reflecting dietary status. A higher HEI score indicates better dietary quality [15].

Regarding physical measurements, height (cm) and weight (kg) data were collected by trained professionals using uniform measurement methods and instruments; waist circumference (cm) referred to the length around the waist at the navel. The waist-to-height ratio was defined as the ratio of waist circumference (cm) to height (cm), and body mass index (BMI) was defined as weight (kg) divided by the square of height (m). The health questionnaire recorded the status of various diseases at the time of the survey, including diabetes (specific definition as described below), hypertension (self-reported doctor-diagnosed hypertension, use of antihypertensive medications, average systolic blood pressure > 140 mmHg, or average diastolic blood pressure > 90 mmHg), chronic obstructive pulmonary disease (COPD) (FEV1/FVC < 0.7, self-reported doctor-diagnosed COPD, use of COPD medications, age over 40 with a history of smoking and chronic). The diagnosis of cardiovascular diseases is determined by trained assessors through self-reported medical diagnoses obtained during individual interviews using standardized questionnaires. Participants were asked, “Has a doctor or other health professional ever told you that you have congestive heart failure/coronary heart disease/angina/myocardial infarction/stroke?” If an individual answered “yes” to any of these questions, they were considered to have cardiovascular disease.

### Definition of TyG related parameters and glucose metabolism status

(1) The TyG index is defined as the natural logarithm of (fasting triglycerides [mg/dL] × fasting glucose [mg/dL]/2).

(2) TyG-WC = TyG × waist circumference (cm).

(3) TyG-BMI = TyG × BMI (kg/m^2^).

(4) WHtR = waist circumference (cm)/height (cm).

(5) TyG-WHtR = TyG × WHtR.

(6) Regarding the classification of glucose metabolism status, in accordance with the criteria established by the American Diabetes Association in 2021 and considering the distribution characteristics in the NHANES database, we categorized glucose metabolism status into the following types [16]

Diabetes (including self-reported doctor-diagnosed diabetes, use of diabetes medications or insulin, or glycated hemoglobin ≥ 6.5%, fasting blood glucose or 2 h OGTT glucose levels ≥ 11.1 mmol/L).

Impaired fasting glucose (not meeting the diagnosis for diabetes, but having fasting blood glucose levels between 5.6 and 6.9 mmol/L and 2 h OGTT glucose levels < 7.8 mmol/L).

Impaired glucose tolerance (not meeting the diagnosis for diabetes, with fasting blood glucose levels < 5.6 mmol/L but 2 h OGTT glucose levels between 7.8 and 11.0 mmol/L).

IFG & IGT: (not meeting the diagnosis for diabetes, with fasting blood glucose levels between 5.6 and 6.9 mmol/L and 2 h OGTT glucose levels between 7.8 and 11.0 mmol/L).

Normal glucose tolerance (fasting blood glucose levels < 5.6 mmol/L and 2 h OGTT glucose levels < 7.8 mmol/L).

### Statistical analyses

The statistical analysis was performed using R software (version 4.3.1), and data extraction and merging were conducted using the nhanesR package (version 0.9.5.0). Multiple imputation was used to handle missing values with the jomo package (version 2.7-6) [17]. Given that the data were derived from a complex sampling survey, weighted calculations were performed using the survey package based on the weighted recommendations from the CDC. For data collected during 1999–2000 and 2001–2002, the weighted coefficient used was wtmec4y multiplied by a factor of 2/10; for data from 2003 to 2019, a weighted coefficient of wtmec2y multiplied by a factor of 1/10 was applied. Continuous variables are presented as weighted means with standard deviation (SD), while categorical variables are expressed as estimated proportions. One-way ANOVA test were used to assess intergroup differences in continuous variables, and chi-square tests were employed for evaluating differences in categorical variables. Categorical variables are reported as absolute frequencies and percentages.

To investigate the association between TyG and its derivatives with the outcome (cardiovascular disease), multivariable logistic regression models were utilized. Furthermore, to understand the correlation between TyG and related parameters with the occurrence of cardiovascular disease across different glucose metabolism levels, we stratified the investigation based on glucose metabolism as a factor and performed interaction analyses to assess potential heterogeneity in associations across subgroups. Additionally, restricted cubic splines and smooth curve-fitting logistic regression models were employed to explore the relationship between TyG and its derivatives with the occurrence of cardiovascular disease. In cases where the relationship was found to be non-linear, logistic regression models were separately conducted on either side of the inflection point to study the association between TyG and its derivatives with the occurrence of cardiovascular disease. Finally, we introduced the TyG index and its related parameters into the classical Framingham cardiovascular model to plot receiver operating characteristic (ROC) curves and analyze the extent of their performance changes in predicting cardiovascular disease in different glucose metabolic states. We utilized the DeLong test to compare the areas under the different ROC curves. The threshold for statistical significance was set at a two-tailed P value of < 0.05.

## Results

### Baseline characteristics of study participants

This study included a total of 14,545 NHANES participants, representing 57 million non-institutionalized U.S. residents. Baseline characteristics of the participants with different glucose metabolism statuses are presented in Table 1. Compared to individuals with normal glucose tolerance, those with diabetes exhibited greater age, weight, BMI, waist circumference, waist-to-height ratio, white blood cell count, uric acid levels, fasting blood glucose, glycated hemoglobin, ALP, BUN, TyG index, TyG-WC, TyG-BMI, TyG-WHtR, higher prevalence of hypertension and cardiovascular disease, and lower height, albumin, eGFR. In comparison to individuals with IFG and IFG & IGT, those with IGT were more likely to have lower height, weight, BMI, waist circumference, waist-to-height ratio, hemoglobin, uric acid, fasting blood glucose, glycated hemoglobin, albumin, BUN, TyG index, TyG-WC, TyG-BMI, TyG-WHtR, smoking rate, and alcohol consumption. In this study, individuals with diabetes had the highest occurrence of cardiovascular disease, followed by those with IFG & IGT, while participants with normal glucose tolerance had the lowest occurrence of cardiovascular disease. All p-values for these comparisons were below 0.05.

**Table 1:** Baseline Characteristics of participants in different glucose metabolism groups

Characteristics	NGT (n = 5281)	IFG (n = 2605)	IGT (n = 584)	IFG&IGT (n = 998)	DM (n = 5077)	*P* value
Age(years), mean(SD)	42.08 (0.36)	48.62 (0.44)	51.28 (0.94)	56.13 (0.65)	59.27 (0.29)	< 0.0001
Sex n(%)						< 0.0001
Male	2267 (43.03)	1646 (62.76)	235 (38.11)	521 (53.73)	2646 (51.15)	
Female	3014 (56.97)	959 (37.24)	349 (61.89)	477 (46.27)	2431 (48.85)	
Height (cm), mean (SD)	168.85 (0.18)	171.30 (0.28)	165.40 (0.47)	168.74 (0.41)	167.58 (0.23)	< 0.0001
Weight (Kg), mean (SD)	77.42 (0.37)	87.44 (0.58)	79.59 (1.00)	89.05 (1.00)	91.56 (0.50)	< 0.0001
BMI (Kg/m^2^), mean (SD)	27.07 (0.12)	29.74 (0.18)	28.96 (0.32)	31.16 (0.32)	32.48 (0.16)	< 0.0001
Waist circumference (cm), mean (SD)	93.41 (0.32)	102.16 (0.41)	98.68 (0.77)	106.14 (0.74)	109.95 (0.37)	< 0.0001
WHtR, mean (SD)	0.55 (0.00)	0.60 (0.00)	0.59 (0.00)	0.63 (0.00)	0.66 (0.00)	< 0.0001
Leukocyte (10^9^/L), mean (SD)	6.48 (0.04)	6.79 (0.06)	6.91(0.12)	7.18 (0.09)	7.44 (0.04)	< 0.0001
Hemoglobin (g/dL), mean (SD)	14.25 (0.03)	14.73 (0.04)	14.21 (0.07)	14.66 (0.06)	14.26 (0.03)	< 0.0001
Platelet (10^9^/L), mean (SD)	247.18 (1.26)	243.91 (1.46)	244.93 (3.30)	247.60 (2.69)	242.95 (1.44)	0.07
Uric acid (mmol/L), mean (SD)	308.49 (1.52)	341.27 (2.04)	327.16 (4.28)	354.21 (3.59)	345.57 (1.97)	< 0.0001
Glucose (mmol/L), mean (SD)	5.11 (0.01)	5.94 (0.01)	5.23 (0.02)	6.10 (0.02)	8.45 (0.06)	< 0.0001
Glycosylated hemoglobin (%), mean (SD)	5.27 (0.01)	5.46 (0.01)	5.42 (0.02)	5.62 (0.02)	7.01 (0.03)	< 0.0001
ALT (U/L), mean (SD)	23.39 (0.22)	27.23 (0.39)	27.07 (1.01)	29.14 (0.78)	27.43 (0.35)	< 0.0001
AST (U/L), mean (SD)	24.41 (0.22)	25.63(0.26)	27.52 (0.81)	27.97 (0.97)	25.74 (0.28)	< 0.0001
Total bilirubin (μmol/L), mean (SD)	12.72 (0.11)	12.54 (0.13)	12.74 (0.30)	12.74 (0.22)	11.44 (0.12)	< 0.0001
ALP(U/L), mean (SD)	63.53 (0.41)	67.71 (0.56)	69.02 (1.47)	70.28 (0.97)	75.19 (0.59)	< 0.0001
Albumin (g/L), mean (SD)	42.93 (0.07)	43.05 (0.08)	42.16 (0.20)	42.67 (0.15)	41.25 (0.08)	< 0.0001
Blood urea nitrogen (mmol/L), mean (SD)	4.45(0.03)	4.85 (0.05)	4.57 (0.12)	4.94 (0.08)	5.71 (0.05)	< 0.0001
eGFR (mL/min/1.73m^2^), mean (SD)	99.51 (0.48)	94.29 (0.50)	91.56 (1.14)	87.96 (0.99)	84.95 (0.49)	< 0.0001
Triglyceride (mmol/L), mean (SD)	1.23 (0.01)	1.49 (0.03)	1.62 (0.07)	1.76 (0.05)	1.94 (0.04)	< 0.0001
LDL (mmol/L), mean (SD)	2.93 (0.02)	3.12 (0.02)	3.12 (0.04)	3.08 (0.04)	2.76 (0.02)	< 0.0001
HDL (mmol/L), mean (SD)	1.47 (0.01)	1.35 (0.01)	1.44 (0.02)	1.31 (0.02)	1.26 (0.01)	< 0.0001
TyG, mean (SD)	8.35 (0.01)	8.70 (0.01)	8.64 (0.03)	8.90 (0.02)	9.21 (0.02)	< 0.0001
TyG-WC, mean (SD)	782.78 (3.17)	890.16 (4.37)	855.53 (8.58)	946.70 (7.47)	1014.51 (4.13)	< 0.0001
TyG-BMI, mean (SD)	226.88 (1.14)	259.30 (1.80)	251.34 (3.23)	278.08 (3.08)	299.75 (1.60)	< 0.0001
TyG-WHtR, mean (SD)	4.64 (0.02)	5.20 (0.03)	5.18 (0.05)	5.61 (0.04)	6.06 (0.02)	< 0.0001
HEI, mean (SD)	53.49 (0.31)	52.52 (0.43)	55.85 (0.85)	53.61 (0.71)	53.60 (0.28)	0.005
Smoking consumption n(%)						< 0.0001
Never smoker	3119 (57.99)	1327 (51.19)	354 (60.31)	502(46.64)	2496 (48.56)	
Former smoker	1002 (20.33)	682 (26.96)	140 (26.00)	334 (36.40)	1729 (34.50)	
Current smoker	1155 (21.69)	593 (21.85)	90 (13.69)	162 (16.96)	847 (16.93)	
Alcohol consumption n(%)						< 0.0001
Never drinking	1396 (22.54)	650 (20.80)	225 (33.37)	369 (31.09)	1978 (36.08)	
Mild drinking	1793 (35.90)	985 (41.09)	184 (34.02)	331 (38.16)	1738 (36.26)	
Moderate drinking	937 (19.22)	401 (17.41)	80 (14.83)	116 (13.11)	547 (12.55)	
Heavy drinking	1155 (22.33)	569 (20.70)	95 (17.79)	182 (17.65)	814 (15.11)	
Race/Ethnicity n(%)						< 0.0001
Non-hispanic white	2374(69.00)	1192 (70.52)	259 (67.53)	486 (73.32)	1872 (63.22)	
Non-hispanic black	1083(10.65)	428 (7.91)	94 (8.65)	127 (6.15)	1186 (12.98)	
Mexican American	772(7.98)	442 (9.18)	106 (10.06)	184 (8.56)	967 (9.28)	
Other	1052(12.37)	543 (12.39)	125 (13.76)	201 (11.97)	1052 (14.52)	
Education n(%)						< 0.0001
Beyond high school	3436 (71.14)	1625 (70.43)	324 (63.89)	575 (66.42)	2803 (64.13)	
High school	672 (9.64)	394 (11.49)	83 (10.64)	148 (11.52)	869 (13.87)	
Below high school	1171 (19.22)	583 (18.09)	177 (25.47)	275 (22.06)	1398 (22.00)	
Family poverty level n(%)						< 0.0001
Poor	1450 (19.79)	721 (18.44)	183 (22.47)	291 (20.06)	1520 (23.35)	
Middle	1816 (35.37)	871 (34.89)	209 (39.45)	362 (37.02)	1968 (41.78)	
Affluent	1661 (44.84)	830 (46.67)	157 (38.08)	266 (42.91)	1178 (34.87)	
COPD n(%)						< 0.0001
No	5099 (96.29)	2462 (94.04)	557 (96.31)	938 (92.80)	4699 (92.85)	
Yes	182 (3.71)	143 (5.96)	27 (3.69)	60 (7.20)	378 (7.15)	
Hypertension n(%)						< 0.0001
No	3912 (76.47)	1603 (62.30)	328 (58.86)	407 (41.74)	1529 (31.86)	
Yes	1369 (23.53)	1002 (37.70)	256 (41.14)	591 (58.26)	3548 (68.14)	
CVD n(%)						< 0.0001
No	5012 (95.57)	2375 (92.39)	524 (91.76)	865 (86.98)	3837 (77.14)	
Yes	269 (4.43)	230 (7.61)	60 (8.24)	133 (13.02)	1240 (22.86)	
Congestive heart failure n(%)						< 0.0001
No	5210 (99.06)	2551 (98.63)	571 (98.18)	953 (95.65)	4624 (92.66)	
Yes	66 (0.94)	51 (1.37)	13 (1.82)	40 (4.35)	421 (7.34)	
Myocardial infarction n(%)						< 0.0001
No	5180 (98.37)	2507 (96.88)	564 (97.14)	935 (94.91)	4544 (90.32)	
Yes	99 (1.63)	97 (3.12)	20 (2.86)	58 (5.09)	519 (9.68)	
Coronary heart disease n(%)						< 0.0001
No	5186 (98.45)	2517 (97.49)	560 (96.55)	930 (93.46)	4547 (90.23)	
Yes	89 (1.55)	80 (2.51)	24 (3.45)	61 (6.54)	479 (9.77)	
Angina pectoris n(%)						< 0.0001
No	5215 (98.99)	2554 (98.54)	566 (97.43)	964 (97.29)	4703 (93.12)	
Yes	58 (1.01)	48 (1.46)	16 (2.57)	28 (2.71)	334(6.88)	
Stroke n(%)						< 0.0001
No	5173 (98.23)	2527 (97.46)	559 (97.03)	960 (96.60)	4662 (92.85)	
Yes	108 (1.77)	78 (2.54)	24 (2.97)	35 (3.40)	406 (7.15)	
Diabetes drugs n(%)						< 0.0001
No	5278 (100.00)	2605 (100.00)	584 (100.00)	997 (100.00)	2112 (43.14)	
Yes	0 (0.00)	0 (0.00)	0 (0.00)	0 (0.00)	2957 (56.86)	
Lipoprotein-lowering drugs n(%)						< 0.0001
No	4819 (90.81)	2174 (82.51)	475 (83.87)	730 (71.13)	2709 (52.38)	
Yes	459 (9.19)	431 (17.49)	109 (16.13)	267 (28.87)	2360 (47.62)	
Antihypertensive drugs n(%)						< 0.0001
No	4436 (85.32)	1939 (74.42)	404 (72.39)	544 (53.97)	1757 (35.79)	
Yes	842 (14.68)	666 (25.58)	180 (27.61)	453 (46.03)	3312 (64.21)	

*BMI* body mass index, *WHtR* waist-to-height ratio, *ALT* alanine aminotransferase, *AST* aspartate aminotransferase, *ALP* alkaline phosphatase, *HEI* Healthy Eating Index, *COPD* chronic obstructive pulmonary disease, *CVD* cardiovascular disease

### Relationship between TyG and its related parameters and the occurrence of cardiovascular disease in different glucose metabolism groups

We constructed multiple logistic regression models to explore the relationship between TyG and its derivative parameters with the occurrence of cardiovascular disease across different glucose metabolism statuses. Ultimately, we found that individuals with different glucose metabolism statuses exhibited varying cardiovascular responsiveness to TyG and its derivative parameters. In the population with normal glucose tolerance, levels of TyG, TyG-WC, and TyG-WHtR were significantly associated with the occurrence of cardiovascular disease (all p < 0.05). For individuals with IFG, a significant association was observed between TyG-BMI (OR: 1.03, 95% CI 1.01–1.05, p = 0.010) and TyG-WHtR (OR: 1.23, 95% CI 1.11–1.35, p = 0.021). Interestingly, in the IGT population, only TyG demonstrated a significant association with the occurrence of cardiovascular disease (OR: 1.94, 95% CI 1.17–3.24, p = 0.016). Among participants with IFG&IGT, TyG-WHtR showed a significant association with cardiovascular disease (OR: 1.42, 95% CI 1.11–1.76, p = 0.028). For individuals with DM, the four indexes all show a significant correlation with the occurrence of cardiovascular disease (all p < 0.05). Furthermore, no significant interactions were observed between the four parameters and glucose metabolism status (all p > 0.05). More information can be found in Table 2.

**Table 2:** Multiple logistic analysis of TyG and its related parameters and the occurrence of cardiovascular disease in different glucose metabolism groups

Characteristics	OR (95% CI)	*P* value	*P* for interaction
TyG			0.131
NGT	1.04 (1.02, 1.07)	** < 0.001**	
IFG	1.05 (0.68, 1.52)	0.785	
IGT	1.94 (1.17, 3.24)	**0.016**	
IFG & IGT	1.02 (0.98, 1.06)	0.240	
DM	1.21 (1.08, 1.37)	**0.010**	
TyG-wc^+^			0.713
NGT	1.03 (1.00, 1.06)	**0.031**	
IFG	1.03 (1.00, 1.06)	0.069	
IGT	1.02 (0.95, 1.10)	0.541	
IFG & IGT	1.01 (0.97, 1.06)	0.557	
DM	1.02 (1.01, 1.03)	**0.023**	
TyG-BMI^+^			0.931
NGT	1.02 (0.99, 1.04)	0.139	
IFG	1.03 (1.01, 1.05)	**0.010**	
IGT	1.03 (0.99, 1.07)	0.105	
IFG & IGT	1.02 (0.97, 1.08)	0.369	
DM	1.02 (1.01, 1.04)	**0.009**	
TyG-WHtR			0.752
NGT	1.06 (1.03, 1.09)	** < 0.001**	
IFG	1.23 (1.11, 1.35)	**0.021**	
IGT	1.08 (0.58, 2.03)	0.803	
IFG & IGT	1.42 (1.11, 1.76)	**0.028**	
DM	1.25 (1.07, 1.47)	**0.002**	

The logistic regression model was adjusted for age, sex, race, smoking, alcohol consumption, poverty and education level, white blood cell count, hemoglobin, platelet count, uric acid, ALT, AST, glomerular filtration rate, HEI, hypertension, and COPD status. In order to better represent the risk effects of disease, we made appropriate adjustments to the units of TyG-WC and TyG-BMI in the regression model. TyG-WC ^+^ represents 1/20* TyG-WC, and TyG-BMI^+^ represents 1/10 *TyG-BMI. For example, in the NGT group, the OR value for TyG-WC is 1.03, indicating that for every increase of 20 in TyG-WC, the risk of cardiovascular disease increases by 3%.

**Fig. 2:**
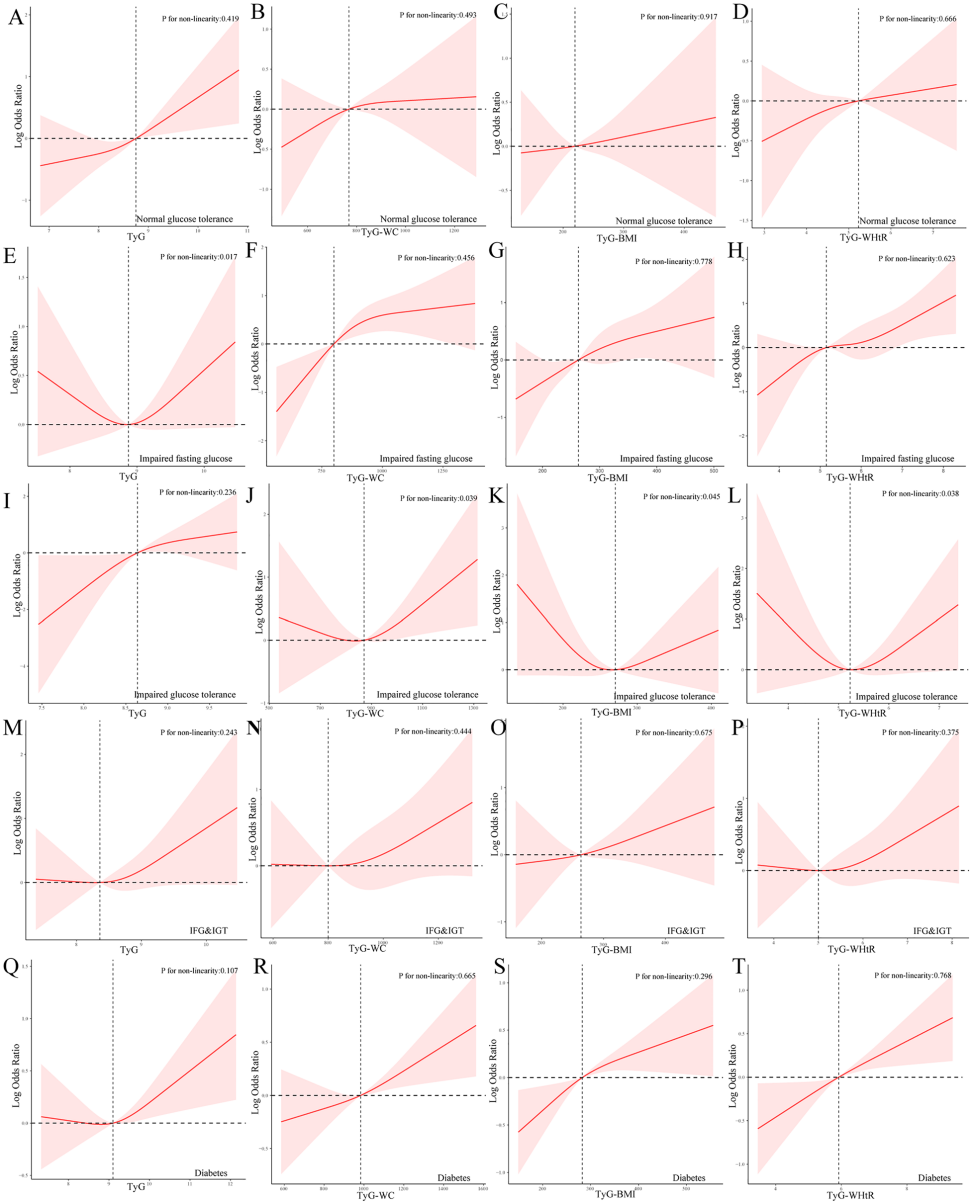
Restricted cubic spline plot of TyG and its derivative parameters with the occurrence of cardiovascular disease under different glucose metabolism statuses. Panels **A**–**D** represent the RCS curves of TyG, TyG-WC, TyG-BMI, and TyG-WHtR in individuals with normal glucose tolerance, respectively. Similarly, panels **E**–**H** represent the IFG individuals, panels **I**–**L** represent the IGT individuals, panels **M**–**P** represent the IFG & IGT individuals, and panels **O**–**T** represent the diabetes individuals

To further analyze the association between TyG and its derivative parameters with the occurrence of cardiovascular disease across different glucose metabolism statuses, we conducted an exploratory analysis using restricted cubic splines (RCS) and smooth curve-fitting logistic regression models, as shown in Fig. 2. We observed curvilinear relationships for the TyG index in the IFG population and for TyG-WC, TyG-BMI, and TyG-WHtR in the IGT population, with p-values for non-linearity < 0.05.

(Models were adjusted for age, sex, race, smoking, alcohol consumption, poverty and education level, white blood cell count, hemoglobin, platelet count, uric acid, ALT, AST, glomerular filtration rate, HEI hypertension, and COPD status. Solid red lines are model estimates, red-shaded regions indicate the 95% confidence intervals)

### Threshold effect analysis

Furthermore, we constructed segmented logistic regression models to study the specific effects before and after the inflection points for the RCS curves that exhibited curvilinear relationships as mentioned earlier. In the IFG population, we observed that when the TyG index was less than 8.88, the cardiovascular risk decreased with increasing TyG index; conversely, when the TyG index was greater than 8.88, this trend reversed. Similarly, in the IGT population, when the TyG-WHtR value was less than 5.23, for each unit increase in TyG-WHtR, the risk of cardiovascular disease decreased by 4% (OR = 0.96, 95% CI 0.93–0.98, p = 0.045); however, when the TyG-WHtR value was greater than 5.23, for each unit increase in TyG-WHtR, the risk of cardiovascular disease increased by 57% (OR = 1.68, 95% CI 11.14–2.12, p = 0.010). TyG-WC and TyG-BMI also demonstrated this trend, with inflection points at 870.90 and 270.45, respectively. More detailed information can be found in Table 3.

**Table 3:** Threshold effect analysis of TyG and its derivative parameters with the occurrence of cardiovascular disease under different glucose metabolism statuses

Characteristics	OR (95%CI)	*P* value
TyG for IFG		
< 8.88	0.86 (0.78, 0.95)	0.016
≥ 8.88	1.43 (1.23, 1.68)	< 0.001
TyG-WC for IGT		
< 870.90	0.95 (0.91, 0.98)	0.031
≥ 870.90	1.08 (1.04, 1.12)	0.018
TyG-BMI for IGT		
< 270.45	0.91 (0.87, 0.96)	0.027
≥ 270.45	1.13 (1.07, 1.24)	0.005
TyG-WHtR for IGT		
< 5.23	0.96 (0.93, 0.98)	0.045
≥ 5.23	1.57 (1.14, 2.12)	0.010

### Incremental analysis of predictive performance of TyG and its derivative parameters

**Fig. 3:**
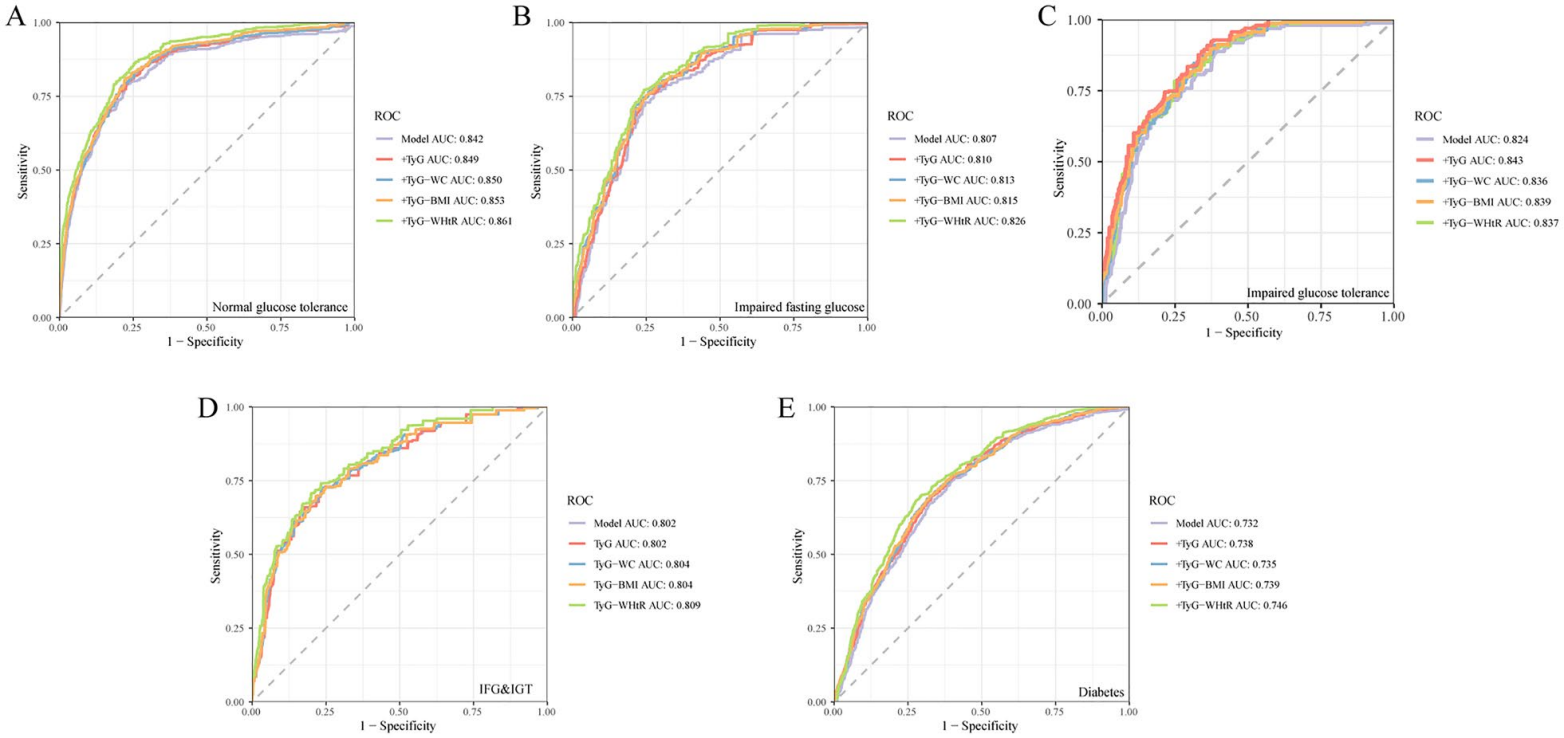
ROC curves of TyG and Its derivative parameters under different glucose metabolism groups. Panel **A** represents the ROC curves of Framingham cardiovascular disease risk model, and adding TyG, TyG-WC, TyG-BMI, and TyG-WHtR additionally to the Framingham model in individuals with normal glucose tolerance, respectively. Similarly, panel **B** represents the IFG individuals, panel **C** represents the IGT individuals, panel **D** represents the IFG & IGT individuals, and panel **E** represents the diabetes individuals

Next, we plotted ROC curves for the Framingham cardiovascular disease risk model, which comprises gender, age, smoking, hypertension, HDL, total cholesterol, and antihypertensive medication use, in different glucose metabolism populations [18]. We then introduced the derived parameters of TyG to construct composite models to understand the changes in predictive performance. We found that introducing different parameters into the traditional model yielded varying increments in different glucose metabolism populations. In the population with normal glucose tolerance, the introduction of TyG-WC and TyG-WHtR into the traditional model showed an increase in predictive performance. Similarly, in the IFG population, the introduction of TyG-WHtR, in the IGT population, the introduction of TyG, and in the DM population, the introduction of TyG-WC and TyG-WHtR all led to a certain degree of improvement in the predictive efficiency of the classical model, with p-values all below 0.05. Among these, the introduction of TyG-WHtR in the NGT, IFG, IFG & IGT and DM groups, and the introduction of TyG in the IGT group maximally enhanced the predictive ability. More details are provided in the Fig. 3 and Table 4 below.

**Table 4:** Results of ROC analysis and DeLong test for different models

Project	NGT		IFG		IGT		IFG & IGT		DM	
	AUC (95% CI)	P value	AUC (95% CI)	P value	AUC (95% CI)	P value	AUC (95% CI)	P value	AUC (95% CI)	P value
Crude model	0.842 (0.836–0.850)	Ref	0.807 (0.791–0.823)	Ref	0.824 (0.801–0.847)	Ref	0.802 (0.794–0.809)	Ref	0.732 (0.726–0.738)	Ref
^+^ TyG index	0.849 (0.841–0.857)	0.284	0.810 (0.794–0.825)	0.788	0.843 (0.824–0.866)	**0.040**	0.802 (0.794–0.810)	0.813	0.738 (0.731–0.743)	0.069
^+^ TyG-WC	0.850 (0.842–0.858)	**0.038**	0.813 (0.797–0.829)	0.575	0.836 (0.813–0.858)	0.798	0.804 (0.796–0.812)	0.561	0.735 (0.728–0.740)	0.391
^+^ TyG-BMI	0.853 (0.844–0.861)	0.019	0.815 (0.798–0.832)	0.335	0.839 (0.816–0.861)	0.492	0.804 (0.796–0.812)	0.469	0.739 (0.732–0.744)	**0.031**
^+^ TyG-WHtR	0.861 (0.853–0.869)	** < 0.001**	0.826 (0.813–0.839)	**0.002**	0.837 (0.811–0.857)	0.699	0.809 (0.803–0.815)	**0.049**	0.746 (0.739–0.753)	**0.017**

The crude model refers to the Framingham cardiovascular disease risk model that incorporates gender, age, smoking, hypertension, HDL, total cholesterol, and antihypertensive medical use

## Discussion

Previous studies have established the TyG index as a risk indicator for atherosclerotic cardiovascular disease (ASCVD) in individuals without ASCVD [19]. Given the clinical relevance of the TyG index and its close association with patient glycemic levels, along with the current lack of evidence in related research, our study was designed to address this gap. Through a large cross-sectional study utilizing the NHANES database, we have unveiled, for the first time, the relationship between TyG and its derived parameters and the occurrence of cardiovascular disease in different glycemic populations, thus providing new evidence for patient risk stratification assessment. By filling this research void, our findings may hold significant implications for the optimization of primary prevention and stratified diagnosis of cardiovascular disease related to TyG parameters. In summary, our study primarily found the following: (1) different associations of TyG and its related parameters with the occurrence of cardiovascular disease in various glucose metabolic states. (2) Curvilinear associations between TyG in the IFG population and TyG-WC, TyG-BMI, and TyG-WHtR in the IGT population and the occurrence of cardiovascular disease. (3) Introduction of the TyG index in the IGT population; and introduction of TyG-WHtR in the NGT, IFG, IFG&IGT, and DM groups can further enhance the predictive performance of classical cardiovascular disease onset models.

Numerous previous studies have indicated that insulin resistance can lead to an increased risk of cardiovascular disease [20]. The hyperglycemic state resulting from insulin resistance damages endothelial function and promotes adverse myocardial remodeling, thereby increasing the risk of atherosclerosis and heart failure [21–23]. Additionally, insulin resistance stimulates vascular smooth muscle cell migration and proliferation through hyperinsulinemia, enhances lipoprotein metabolism, and promotes cardiovascular disease [24, 25]. Additionally, heightened insulin levels contribute to an increase in sympathetic nervous system activity and cardiac output, resulting in hypertension and vascular harm [26]. Furthermore, due to decreased cellular responsiveness to insulin, fat is not effectively transported and utilized, resulting in elevated blood lipid levels [27, 28]. Elevated lipid levels have been associated with endothelial dysfunction, heightened platelet aggregation, and augmented inflammatory response, consequently fostering the onset of cardiovascular disease [28].

Different glucose metabolic states involve distinct pathophysiological mechanisms. IFG is distinguished by concurrent hepatic insulin resistance and early insulin secretion anomalies, culminating in elevated fasting hepatic glucose production and subsequent fasting hyperglycemia. Conversely, IGT is marked by nearly normal hepatic insulin sensitivity, substantial muscle insulin resistance, and late insulin secretion deficiencies, resulting in prolonged postprandial hyperglycemia, while diabetes signifies systemic insulin resistance reaching a decompensated stage [29, 30]. Furthermore, the Insulin Resistance Atherosclerosis Study has demonstrated significantly different lipid profiles between IFG and IGT [31]. Mary et al. reported that globally, the prevalence of IGT and IFG among adults aged 20–79 years was 9.1% and 5.8%, respectively [32]. Due to insufficient research, there remains a severe lack of clinical understanding of different glucose metabolic states on a global scale [32, 33]. Therefore, several researchers have advocated for further refinement of the management of prediabetic patients [34–36].

The TyG index, composed of TG and FBG, has been recognized as a simple yet efficient marker of insulin resistance with significant value in various fields [37, 38]. We found that the OR of TyG with CVDs remained significantly different in the IGT group compared to other groups. Notably, introducing the TyG index into the classical Framingham cardiovascular model significantly improved the model’s area under the curve [0.843 (0.824–0.866) vs 0.824 (0.801–0.847; P = 0.04)]. One possible explanation for this observation is that IGT represents a prediabetic state, serving as an intermediary phase between normal glucose metabolism and diabetes. This stage may signify a crucial period of rapid escalation in cardiovascular risk, as it reflects escalating metabolic disturbances that have not yet reached the typical threshold of diabetes [39]. Therefore, the TyG index during the IGT stage may more accurately capture the cardiovascular disease risk during this transitional phase. However, the specific mechanisms underlying this phenomenon remain unclear, warranting further in-depth research in the future.

To our knowledge, there are few studies that have examined the association between the TyG index, along with its derived parameters, and the risk of CVD among individuals with NGT. However, our research has identified significant associations between the TyG index, TyG-WC, and TyG-WHtR indices and the incidence of CVD in this special group. Previous studies, such as that by Cai et al., found a positive correlation between the TyG index and the risk of arterial stiffness within populations exhibiting normal glucose metabolism [40]. Additionally, research conducted by Guo et al. has shown that a high TyG index is significantly associated with impaired cardiovascular fitness in young, non-diabetic individuals, where mediation analysis indicated that part of the adverse effects could be attributed to its impact on blood pressure and abdominal obesity [41]. Regrettably, to date, studies have primarily focused on observing the statistical significance of these associations, with the underlying mechanisms still requiring further elucidation. Despite this, our study contributes valuable insights, particularly as we found that the inclusion of anthropometric indicators reflective of abdominal obesity, such as WC and WHtR, continues to demonstrate a significant effect, indirectly corroborating Guo et al.’s hypothesis. This underscores the importance of considering the TyG index and its derivatives as potential markers for cardiovascular risk, even in individuals with ostensibly normal glucose metabolism.

Parameters derived from TyG have also demonstrated excellent early discriminative ability in non-alcoholic fatty liver disease and metabolic syndrome [13]. However, their role in different glycemic populations has received little attention. We observed a U-shaped correlation between all three derived TyG parameters in IGT and TyG index in IFG and cardiovascular risk. We found that maintaining TyG-WC at 870.90 or TyG-BMI at 270.45, and TyG-WHtR at around 5.23, results in lower cardiovascular risk for the IGT group. Similarly, keeping TyG at 8.88 can minimize cardiovascular risk for IFG. One possible explanation is that IGT exhibits distinct whole-body fat distribution characteristics compared to other glucose metabolic states [31, 42]. Consistent with prior research, our study also found that individuals with IGT have smaller waist circumferences and lower BMIs than those with IFG and diabetes [43, 44]. Considering that TyG-derived parameters are a combination of TyG and body measurement indicators, these differences in fat distribution may account for the varying predictive efficacy of TyG and its derived parameters in the IGT population. Furthermore, the U-shaped correlation between TyG index in IFG may be attributed to the low TyG index levels in the IFG group, indicating low triglyceride levels, which previous studies have linked to increased risk of hemorrhagic stroke and cardiovascular death in heart failure patients [45, 46]. Therefore, it is crucial for individuals with IFG to maintain appropriate TyG index levels. The disappearance of the U-shaped correlation of TyG-derived parameters in the IFG group may be explained by the introduction of body measurement indicators weakening this correlation However, it is worth noting that while this study identified these U-shaped associations and determined their thresholds, further validation of these thresholds is needed to increase evidence support in the future.

Of particular significance, in the NGT, IFG, IFG&IGT, and DM groups, TyG-WHtR demonstrated superior predictive capability for cardiovascular disease, outperforming other TyG-derived parameters. Specifically, for every unit increase in TyG-WHtR, the cardiovascular risk increased by 6% in the NGT group (95% CI 1.03, 1.09; P < 0.001), 23% in the IFG group (95% CI 1.11, 1.35; P = 0.021), 42% in the IFG&IGT group (95% CI 1.11, 1.76; P = 0.028), and 25% in the DM group (95% CI 1.07, 1.47; P = 0.002). Furthermore, the introduction of TyG-WHtR significantly improved the efficiency of cardiovascular prediction models in these populations (P < 0.05 for all). The findings can be elucidated through the distinct contributions of WC, BMI, and WHtR in evaluating obesity. BMI, being a measure based on height and weight, cannot distinguish between abnormal fat accumulation and general obesity. Additionally, the metabolism of adipose tissue cannot be determined by BMI alone [47]. Moreover, abdominal obesity encompasses both subcutaneous and visceral fat accumulation [48]. Early research has shown that the excessive accumulation of visceral fat is more closely linked to cardiovascular risk, given its heightened secretion of inflammatory and multiple cytokines [49, 50]. WC and WHtR serve as markers of visceral obesity [51]. Due to its adjustment for height, WHtR serves as a superior indicator of abdominal obesity compared to both BMI and WC. When integrated with the TyG index, it more accurately mirrors the impact of abdominal obesity and insulin resistance on cardiovascular disease. [52]. Interestingly, we conducted a separate analysis of the Non-Hispanic White subgroup to investigate racial specificity. We found a significant correlation between the TyG index and cardiovascular disease risk in IGT patients, as well as a significant correlation between TyG-WHtR and cardiovascular disease risk in NGT, IFG, IFG & IGT, and DM patients. Detailed data can be found in Supplementary Table 1. Previous research from China and Thailand has confirmed that TyG-WHtR is the most practical biomarker for predicting the risk of hypertension onset [53, 54]. However, Miao et al.’s study suggested that the predictive efficacy of TyG-WHtR for cardiovascular risk was not as strong as that of TyG-WC in the Asian population. [55]. This inconsistency can be understood considering the influence of different glucose metabolic states and substantial variations in ethnicity, diet, and exercise habits. Thus, further validation of our study’s findings in additional countries or regions is warranted in the future.

### Strengths and limitations

The strengths of this study are primarily demonstrated in the following aspects. Firstly, we designed a large cross-sectional study to analyze the association between the TyG index and its derived parameters and cardiovascular disease in different glucose metabolic states. The sample size of this study is sufficiently large to represent 57 million non-institutionalized civilians in the United States, making the results highly reliable and valuable as a reference. Secondly, by introducing body measurement indicators to enhance the TyG index, we overcame its original limitations and significantly improved its relationship with cardiovascular disease, thereby enhancing the efficiency of the classic Framingham cardiovascular prediction model. Considering that the calculation of TyG only requires triglycerides and fasting blood glucose, and body measurement indicators are easily obtainable, the results of this study are highly suitable for large-scale epidemiological investigations, with significant public health implications. However, this study also has some limitations that should not be overlooked. Firstly, as a retrospective study, we cannot confirm whether there is a causal relationship between the variables investigated. Secondly, we only measured the values of various indicators once, and the continuous longitudinal changes in these parameters and their correlation with clinical outcomes remain unknown. Additionally, this study did not investigate the differences between type 1 and type 2 diabetes in detail, which may have led to some bias in the final results. Fourthly, this study only included the American population, and considering the differences in ethnicity, diet, exercise, and sleep habits, the results of this study should be further validated in more countries or regions. Lastly, some potential confounders may not have been fully considered. Further research is needed to validate the results obtained in this study.

## Conclusions

In conclusion, this study found that in different glucose metabolic states, the TyG index and its related parameters have different associations with the occurrence of cardiovascular disease. In the NGT, IFG, IFG & IGT, and DM groups, TyG-WHtR showed a significant association with cardiovascular disease, while the TyG index showed a significant association in individuals with IGT. Correspondingly, introducing the aforementioned parameters into the Framingham cardiovascular risk model can further improve the predictive performance of the model. The results of this study provide new insights into the relationship between the TyG index and its derived parameters in different glucose metabolic states and the risk of cardiovascular disease, offering important reference value for future clinical practice and research.

### Supplementary Information

Supplementary Material 1.

## Data Availability

All datasets used in this study are publicly available on the NHANES website. (https://wwwn.cdc.gov/nchs/nhanes/AnalyticGuidelines.aspx).
