# Supplementary material for: The association between triglyceride-glucose index and related parameters and risk of cardiovascular disease in American adults under different glucose metabolic states
Source: Diabetol Metab Syndr. 2024 May 17;16:102. doi: 10.1186/s13098-024-01340-w (PMC11100199; doi:10.1186/s13098-024-01340-w)
Supplement: Supplementary file 1 — Supplementary Material 1. [file 13098_2024_1340_MOESM1_ESM.docx]

**Supplementary Material**

**The Association Between triglyceride-glucose index and Related Parameters and** **Risk of Cardiovascular Disease in American Adults Under Different Glucose Metabolic States**

Yuansong Zhuang^1^, Liliang Qiu^2^ Dongjian Han^1^, Zhentao Qiao^3^, Fuhang Wang^1^, Qingjiao Jiang^1^, Quanxu An^1^, Yuhang Li^1^, Jiahong Shangguan^1^, Xuanye Bi^1^, Deliang Shen^1*^

1. Cardiology Department, First Affiliated Hospital of Zhengzhou University, Henan, China.

2. Department of Respiratory Medicine, First Affiliated Hospital of Zhengzhou University, Henan, China.

3.Department of Vascular and Endovascular Surgery, First Affiliated Hospital of Zhengzhou University, Henan, China.

* Correspondence: Deliang Shen, Cardiology Department, First Affiliated Hospital of Zhengzhou University, Henan, China. Tel:+86-15038335158. E-mail: dlshen@zzu.edu.cn

**Table 1** Multiple logistic analysis of TyG and its related parameters and the occurrence of cardiovascular disease in different glucose metabolism groups in Non-Hispanic White group.

| Characteristics | OR (95% CI) | *P* value | *P* for interaction |
| --- | --- | --- | --- |
| TyG |  |  | 0.122 |
| NGT | 1.17(0.94, 1.46) | 0.159 |  |
| IFG | 1.26(0.83, 1.92) | 0.274 |  |
| IGT | 1.88(1.06, 3.32) | **0.031** |  |
| IFG&IGT | 1.04(0.75, 1.44) | 0.815 |  |
| DM | 1.20(0.53, 2.69) | 0.657 |  |
| TyG-wc^+^ |  |  | 0.188 |
| NGT | 1.03(0.98, 1.14) | 0.098 |  |
| IFG | 1.03(0.91, 1.17) | 0.632 |  |
| IGT | 1.06(0.96, 1.17) | 0.236 |  |
| IFG&IGT | 1.02(0.95, 1.08) | 0.637 |  |
| DM | 1.09(0.90, 1.32) | 0.248 |  |
| TyG-BMI^+^ |  |  | 0.327 |
| NGT | 1.04(0.97, 1.11) | 0.317 |  |
| IFG | 1.02(0.99, 1.05) | 0.113 |  |
| IGT | 1.04(0.99, 1.08) | 0.081 |  |
| IFG&IGT | 1.03(0.98, 1.08) | 0.154 |  |
| DM | 1.01(0.94, 1.09) | 0.784 |  |
| TyG-WHtR |  |  | 0.889 |
| NGT | 1.03(1.01, 1.06) | **0.049** |  |
| IFG | 1.24(1.02, 1.52) | **0.031** |  |
| IGT | 1.25(0.94, 1.65) | 0.128 |  |
| IFG&IGT | 1.35(1.04, 1.76) | **0.026** |  |
| DM | 1.05(1.02, 1.08) | **0.002** |  |

(The logistic regression model was adjusted for age, sex, race, smoking, alcohol consumption, poverty and education level, white blood cell count, hemoglobin, platelet count, uric acid, ALT, AST, glomerular filtration rate, HEI, hypertension, and COPD status. In order to better represent the risk effects of disease, we made appropriate adjustments to the units of TyG-WC and TyG-BMI in the regression model. TyG-WC+ represents 1/20 * TyG-WC, and TyG-BMI+ represents 1/10 * TyG-BMI. For example, in the NGT group, the OR value for TyG-WC is 1.04, indicating that for every increase of 20 in TyG-WC, the risk of cardiovascular disease increases by 4%.)
